# Elaborate Details, Hidden Surprises

**DOI:** 10.3201/eid2307.AC2307

**Published:** 2017-07

**Authors:** Byron Breedlove, Terence Chorba

**Affiliations:** Centers for Disease Control and Prevention, Atlanta, Georgia, USA

**Keywords:** art science connection, emerging infectious diseases, art and medicine, about the cover, Fabergé Imperial eggs, public health, intracellular pathogens, microbes, viruses, bacteria, fungi, protozoa, World War I, Imperial Red Cross Easter Egg, elaborate details, hidden surprises

**Figure:**
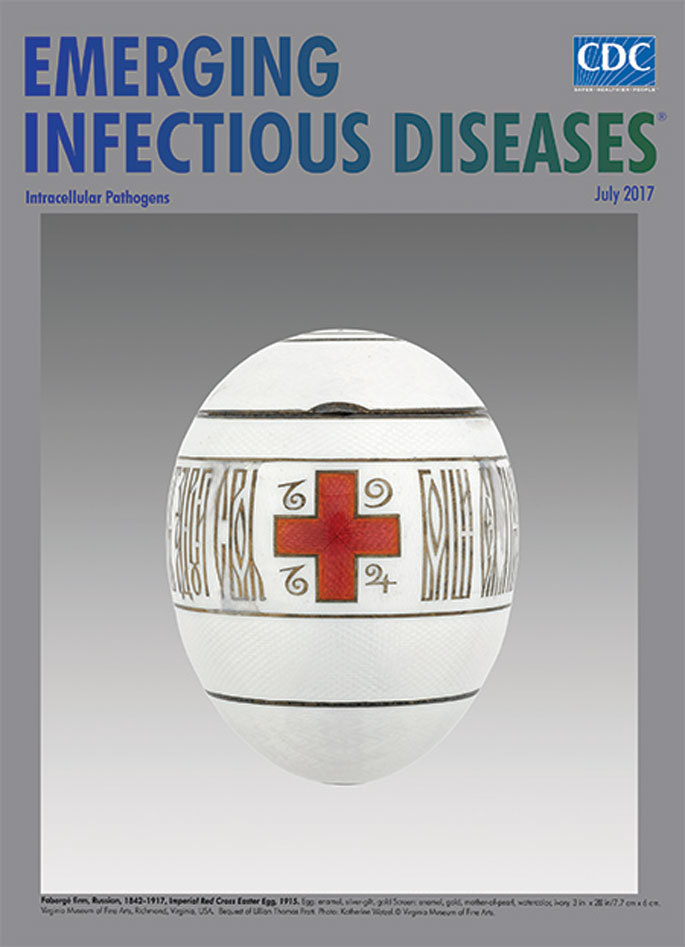
**Fabergé firm, Russian, 1842–1917, *Imperial Red Cross Easter Egg*, 1915. Egg: enamel, silver-gilt, gold Screen: enamel, gold, mother-of-pearl, watercolor, ivory, 3 in x 2 3/8 in/7.7 cm x 6 cm.** Virginia Museum of Fine Arts, Richmond, Virginia, USA. Bequest of Lillian Thomas Pratt. Photo: Katherine Wetzel, © Virginia Museum of Fine Arts.

In 1885, Russian Emperor Alexander III commissioned the first Imperial Easter egg from the House of Fabergé as a gift for his wife, Maria Feodorovna. Upon Alexander’s death in 1894, his son, Emperor Nikolai II, continued this Imperial family tradition, annually requesting one egg for his mother and another for his wife. The skilled artisans who created these treasures worked under the auspices of master craftsman Peter Carl Fabergé, head of the firm that still bears his family name.

Fabergé operated with creative freedom, and his craftsmen had a treasure chest of riches at their disposal. Diamonds, pearls, sapphires, rubies, gold, guilloché enamel, aquamarine, lapis lazuli, ivory, mother of pearl, and platinum were commonly used embellishments. Each of these unique 50 *objets d’art* created during 1885–1916 shared a single feature: a concealed surprise. These sequestered miniature wonders include a rotating globe, a replica of Gatchina Palace, a four-leaf clover, a gold watch, Peter the Great’s monument on the Neva River, miniature portraits, a mechanical swan, an 18th century carriage that took 14 months to complete, and a jeweled elephant automaton (rediscovered in 2015 and reunited with its egg in 2017).

Fabergé expert Géza von Habsburg describes Fabergé eggs as being “the absolute summit of craftsmanship. They are unbelievably made. They were the sort of apogee of what Fabergé was able to do, and he lavished everything he could on them.” The whereabouts of 43 of the 50 Imperial Fabergé eggs are known, and 5, including the Imperial Red Cross Easter Egg featured on this month’s cover, are found in the Virginia Museum of Fine Arts.

This egg, created in 1915 during World War I, honors the efforts of the Empress Alexandra Feodorovna, who served as head of the Russian branch of the International Red Cross. Feodorovna and her older daughters had trained to become nurses, and she had the Winter Palace set up to function as a makeshift hospital for the growing number of wounded soldiers. Because Fabergé recognized that the mood in Russia was growing more solemn and grave as the war progressed, he focused on the royal family’s service instead of showcasing its lavishness during this time of wartime austerity.

The Fabergé Research Site offers this description for the egg, which was created by workmaster Henrik Wigström: “Opalescent white guilloche enamel covers a chased silver ground on this egg. Two opposing red enamel crosses bear the dates 1914 and 1915. A Russian inscription, in stylized gold enamel script in a band around the egg reads: ‘Greater love hath no man than this, that a man lay down his life for his comrades.’ On the top of the egg is the crown and monogram of the Dowager Empress Marie in silver, while at the bottom is a six-petal rosette.”

Concealed inside this egg is a hinged, folding screen that displays 5 miniature portraits of the tsar’s mother, sister, daughters, and cousin wearing Red Cross uniforms (Figure). Each portrait is encased in white enamel, mounted in gold, and backed with mother of pearl. The screen is signed by craftsman Vassilii Zuiev.

**Figure:**
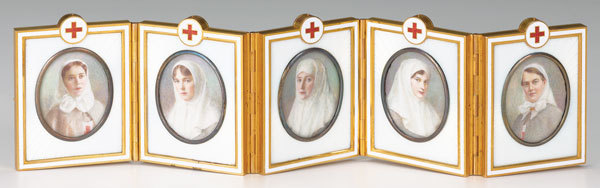
Concealed inside this egg is a hinged, folding screen that displays 5 miniature portraits of the tsar’s mother, sister, daughters, and cousin wearing Red Cross uniforms. Each portrait is encased in white enamel, mounted in gold, and backed with mother of pearl. The screen is signed by craftsman Vassilii Zuiev.

At this time, the fall of the Romanov dynasty was nearing. The Imperial Fabergé eggs, festooned with jewels and fine metals and containing bejeweled hidden surprises, remain as delicate reminders.

Within microbiological realms, intracellular pathogens offer an analogous melding of elaborate detail and hidden surprises. Honed through evolution rather than hours logged on an artisan’s workbench, intracellular pathogens comprise an ancient, diverse group of microbes that by dint of moving into the intracellular environment have mastered living within the hostile realm of host macrophages.

Intracellular pathogens are classified into two groups: obligate and facultative. Obligate intracellular pathogens cannot survive outside of their host cells and include viruses, some bacteria (*Chlamydiae*, *Rickettsia* spp., *Coxiella burnetti*, *Mycobacterium leprae*), and protozoa. Facultative intracellular pathogens survive and replicate outside of their host cells and include many bacteria (*Legionella pneumophila*, *Rickettsia rickettsii*, *Mycobacterium tuberculosis*, *Listeria monocytogenes*, *Salmonella* spp., *Shigella* spp., invasive *Escherichia coli*, *Neisseria* spp., and *Brucella* spp.) and fungi (*Histoplasma capsulatum*).

Intracellular pathogens sequestered within the walls of their host cells commonly cause granulomatous lesions and are not delightful surprises, unlike the surprises ensconced inside the Fabergé Imperial Eggs. In addition to those previously noted, a partial roll call of intracellular pathogens includes *Yersinia pestis*, *Cryptococcus neoformans*, *Burkholderia pseudomallei*, and *Plasmodium* spp. Such pathogens are clearly not rarities, cause a wide range of infections in animals, and are major contributors to human morbidity and mortality globally.

During World War I and its wake, an estimated 16 to 22 million military combatants and civilians died. Professor Francis Cox of Gresham College has noted the difficulty of obtaining accurate figures and states that “it is almost impossible to make any meaningful comparisons between the numbers of those who died in combat and those who died from disease as a direct result of combat.” The Red Cross emblazoned on this 1915 Fabergé Imperial egg reminds us that healing and medicine are intrinsically intertwined with conflict. As Professor Cox has written, “. . . all over the world in every combat zone, big or small, diseases still have the potential to hold the upper hand and . . . we are complicit in their survival and spread.”

## References

[1] Casadevall A. Evolution of intracellular pathogens. Annu Rev Microbiol. 2008;62:19–33. 18785836 10.1146/annurev.micro.61.080706.093305

[2] Cox F. The First World War: disease, the only victor (transcript of lecture March 10, 2014) [cited 2017 Jun 7]. https://www.gresham.ac.uk/lectures-and-events/the-first-world-war-disease-the-only-victor

[3] Fabergé Research Site. Red Cross portraits egg (1915) [cited 2017 May 18]. http://fabergeresearch.com/eggs-faberge-imperial-egg-chronology/#redcrossportraitsegg

[4] Public Broadcast System. Fabergé eggs [cited 2017 May 18]. http://www.pbs.org/treasuresoftheworld/faberge/flevel_1/f2_featured_eggs.html

[5] Raoult D, Brouqui P. Intracellular location of microorganisms. In: Raoult D, editor. Antimicrobial agents and intracellular pathogens. Boca Raton (FL): CRC Press; 1995. p. 40–56.

[6] Ray K, Marteyn B, Sansonetti PJ, Tang CM. Life on the inside: the intracellular lifestyle of cytosolic bacteria. Nat Rev Microbiol. 2009;7:333–40. 19369949 10.1038/nrmicro2112

[7] Stengle J. Faberge egg reunited with its missing ‘surprise’ in Texas [cited 2017 Jun 1]. https://phys.org/news/2017-04-faberge-egg-reunited-texas.html

[8] Warde-Aldam D. Easter egg hunt: the Third Imperial Fabergé Easter egg has resurfaced. Apollo. April 10, 2014 [cited 2017 May 18]. https://www.apollo-magazine.com/easter-egg-hunt-third-imperial-faberge-easter-egg-resurfaced/

